# Patients' expectations and experiences of stem cell therapy for the treatment of knee osteoarthritis

**DOI:** 10.1111/hex.13113

**Published:** 2020-08-14

**Authors:** Lucinda Kenihan, Lauren McTier, Nicole M. Phillips

**Affiliations:** ^1^ Melbourne Stem Cell Clinic Box Hill VIC. Australia; ^2^ Centre for Quality and Patient Safety Research School of Nursing and Midwifery Deakin University Geelong VIC. Australia

**Keywords:** knee osteoarthritis, patient expectations, patient experience, stem cell therapy

## Abstract

**Background:**

Stem cell therapy is a novel treatment option for people living with osteoarthritis. Research investigating stem cell therapy for this debilitating condition has predominantly involved the pathogenesis of the cells and efficacy of the treatment. There is little understanding of patients' expectations and experiences of stem cell therapy treatment.

**Objective:**

To explore the expectations and experiences of people undergoing stem cell therapy for the treatment of knee osteoarthritis.

**Design:**

An exploratory, descriptive, qualitative study using semi‐structured interviews was conducted.

**Setting and participants:**

Participants were recruited into two groups: (a) Expectations Group (*n* = 15); the expectations of stem cell treatment were explored with participants that were yet to commence stem cell therapy. (b) Experiences Group (*n* = 15); the experiences of stem cell therapy were explored with participants 12 months after their initial stem cell treatment. Transcripts were analysed using thematic analysis to identify themes in both groups.

**Results:**

Themes for the Expectations Group were active involvement in the treatment; treatment will improve symptoms; and benefits of treatment outweigh the risks. Themes for the Experiences Group were symptoms of treatment; satisfaction with treatment; and anticipation of further improvement.

**Discussion and conclusions:**

The findings are the first qualitative study to represent patients' perspective on expectations and experiences of stem cell therapy for knee osteoarthritis. They provide insight into the potential areas for improvement within this field to aid patients' preparation and approach to the treatment, promoting patient‐centred care.

## 1 INTRODUCTION

In Australia, approximately 2.2 million people suffer from osteoarthritis,^1^ and 50% are aged 65 years and over.^2^ It is forecast that by 2030, the number of people living with osteoarthritis in Australia will be 5.4 million,^3^ equating to a 157% increase in the prevalence of the disease over the next 10 years. Moreover, the cost to the welfare and health system to address this disease is predicted to grow by $150 million each year.^3^ Worldwide, arthritis is considered to be in the top four leading causes of disability.^4^, ^5^ With prominent ageing populations, it is predicted that the number of people suffering with arthritis globally will double by 2020.^6^ While osteoarthritis can affect many joints in the body, it often affects the knee.

Traditional treatments for osteoarthritis of the knee include conservative management techniques such as application of ice, compression and exercise,^7^ pharmacological pain management, arthroscopy and knee arthroplasty and/or knee replacement.^4^ These treatment strategies focus on pain reduction and management of symptoms. They do not offer disease modifying results.^4^ In some instances, evidence has even shown few efficacious results. Arthroscopy procedures have been found to provide inconsequential value/benefit to patients with osteoarthritis due to the surgery not addressing its main effect, articular degeneration.^7^, ^8^, ^9^, ^10^, ^11^ Furthermore, it has been reported that an estimated one‐quarter of total knee replacements are performed on inappropriate candidates with 15‐30% of patients reporting dissatisfaction with the treatment.^2^, ^12^, ^13^, ^14^ Up to 54% of patients report residual symptoms and functional problems following knee replacement.^13^ Total knee replacements also have risk of deformity and surgical failure.^4^, ^15^ Some post‐operative complications include myocardial infarction, deep vein thrombosis, deep and superficial wound infections^16^, ^17^ pulmonary embolus and trapped nerve.^18^ Consequently, total knee replacements are viewed as a last resort and/or measurably delayed as a treatment for osteoarthritis.^7^, ^19^

People with osteoarthritis of the knee, having exhausted traditional therapies, wanting to delay them, or not being eligible for them, may seek out stem cell therapy to resolve their symptoms. Stem cell therapy is a novel treatment which focuses on disease modification rather than management. Initial research suggests stem cell therapy is a safe and effective treatment for osteoarthritis of the knee.^20^, ^21^, ^22^, ^23^ The therapy is costly to the patient and requires adherence to treatment recommendations in order to optimize treatment outcomes. The patient perspective of stem cell treatment has been given little attention in published research.

The aims of this study were to explore the expectations of people that had elected to receive stem cell therapy and the experiences of people that had received stem cell therapy for the treatment of knee osteoarthritis. The principle research questions were as follows:

1. What are the expectations of people having stem cell therapy for osteoarthritis of the knee?
2. What are the experiences of people having stem cell therapy for osteoarthritis of the knee?

Advancing knowledge in these areas can inform patient‐centred care by better preparing patients for this treatment, where patient expectations align with possible treatment outcomes, subsequently enhancing their experience.

## 2 METHODS

An exploratory, descriptive, qualitative study using naturalistic inquiry via semi‐structured interviews was conducted.^24^, ^25^, ^26^ This design allowed for in‐depth descriptive accounts of participants' expectations or experiences of stem cell therapy.^27^, ^28^ Ethics approval to conduct this study was granted by the Human Research Ethics Committee at Deakin University.

### 2.1 Setting

The private Stem Cell Centre where the study was conducted has been operational for nearly six years and primarily treats patients with osteoarthritis of the knee. Approximately 35 patients with varying severity of osteoarthritis are treated each month at the centre. The treatment process is individualized for optimal therapeutic outcomes.

**FIGURE 1:**
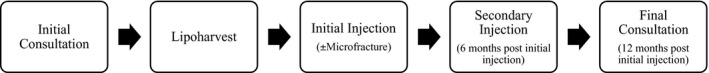
Treatment trajectory for patients receiving stem cell therapy for osteoarthritis of the knee

Figure 1 outlines the treatment trajectory for patients receiving stem cell therapy for osteoarthritis of the knee. A lipoharvest is the procedure for extracting the adipose tissue from patients. This method is used to collect a sample of the patient's stem cells for culturing. The initial injection procedure occurs after the lipoharvest, once the stem cells have been cultured, and pertains to the first dose of the patient's stem cells being reintroduced back into the affected joint. Micro‐fracture surgery may occur immediately prior to the initial injection of stem cells and aims to stimulate and assist with healing at a chondral defect site by aggravating the area. Only a small percentage of patients (5%) undergo micro‐fracture surgery prior to the initial injection at the Stem Cell Centre.

### 2.2 Participants

Participants were recruited from a single site in Melbourne, Victoria, Australia, by a convenience sampling method. Patients attending the Private Stem Cell Clinic for treatment of osteoarthritis of the knee that were aged over 18 years and spoke English were invited to participate.

Thirty participants were recruited into two separate groups:

1. Expectations Group (*n* = 15) had consented to stem cell therapy but were yet to commence treatment. Their expectations of stem cell therapy were explored.
2. Experiences Group (*n* = 15) had completed treatment (12 months after their initial treatment). Their experiences of stem cell therapy were explored.

The student researcher was employed as a nurse at the private Stem Cell Centre two days per week. The student may have provided care to potential participants for their initial stem cell treatment at least six months prior to the study commencement so an initial invitation to participate in the study was undertaken by the Stem Cell Clinic receptionist who was not involved in the research. Verbal consent was gained by the receptionist to be approached by the student investigator who discussed the purpose of the study and provided a plain language statement to potential participants. Participants were made aware that their involvement was completely voluntary and did not impact their treatment. The role of the student investigator conducting the study as part of an Honours degree was explained and it was reinforced that this was separate from the student investigator's employment at the centre.

Written consent was obtained from all participants. Recruitment was conducted over a 6‐month period until 15 participants were recruited for each the Expectations Group and the Experiences Group (total of 30 participants). Participants in the Expectations Group were recruited after their lipoharvest, prior to their initial injection. Participants in the Experiences Group were recruited following their final consultation, 12 months after their initial injection. This was because effects of stem cell treatment are most likely to be experienced by patients at a 12‐month interval, after initial injection, compared to earlier follow‐up.^5^, ^20^, ^29^ The sample size was considered appropriate to discover and understand the study phenomenon due to its novelty, specificity^30^, ^31^, ^32^ and reliability on experiential relevance.^33^

### 2.3 Data collection

Individual participant semi‐structured interviews, up to 60 minutes in duration, were conducted over the telephone. Interviews were conducted via telephone in order to further mitigate the impact of the student investigator's employment at the Stem Cell Centre. All audio files were transcribed verbatim and checked twice against the audio file to eradicate any errors. Semi‐structured interview templates were developed by the research team considering the research aims and questions. The list of questions was open‐ended to allow the participants to elaborate and express their feelings and not be influenced by interviewer input.

Throughout each interview, field notes were written. The student researcher's assumptions and perceptions of the interview were scribed, subsequently informing participant meaning. The field notes also included uncommon phrases and expressions said by participants to ensure correct transcription of the audio was achieved. Separate journaling throughout the data analysis indicating how themes emerged and the student investigator's personal thoughts of the process was also undertaken in keeping with consistency and trustworthiness.^34^ These entries included a decision/audit trail and expression of difficulties the student investigator encountered.

### 2.4 Data analysis

At the completion of each interview, member checking occurred via two processes to ensure trustworthiness, consistency, confirmability and truth value of the findings.^34^, ^35^ The first process entailed repeating a summary of the points raised back to the participant, at the end of the interview, giving them the opportunity to disagree and/or restate any aspect of the interview. Participants were also invited to review a summary of their transcript, seven to ten days following their interview. Of the 30 participants, none requested a repeat interview.

The qualitative data analysis was undertaken using thematic analysis as described by Braun and Clarke.^36^ On‐going critical review of the themes was conducted individually by all research members comparing them against the transcripts in order to appraise their relevance, uphold credibility and truth value. Peer discussion also occurred throughout the development of themes among the research team.

## 3 RESULTS

Similar participant characteristics were noted in both the Expectations and Experiences Groups (Table 1). The majority of participants were male (70%) with a mean age of 56 years. Most participants were married, employed and had an education level equivalent to or greater than successful completion of high school. Of the 15 participants that had completed treatment (Experiences Group), three (20%) received micro‐fracture surgery prior to the initial injection of stem cells.

**TABLE 1:** Participant characteristics (*N* = 30)

Characteristics	Expectations group (*n* = 15)	Experiences group (*n* = 15)
Age mean (SD)	56.5 (10.7)	55.5 (17.3)
Male *n* (%)	11 (73.3)	10 (66.7)
Education *n* (%)		
VCE^a^ not completed	4 (26.7)	2 (13.3)
VCE or equivalent	3 (20.0)	3 (20.0)
TAFE or trade	0 (0.0)	1 (6.7)
Bachelors or diploma	6 (40.0)	5 (33.3)
Post‐graduate or PhD	2 (13.3)	4 (26.7)
Employed *n* (%)	11 (73.3)	12 (80.0)
Marital status *n* (%)		
Single	3 (20.0)	3 (20.0)
De facto	2 (13.3)	1 (6.7)
Married	10 (66.7)	11 (73.3)
Home residence in Melbourne *n* (%)	9 (60.0)	4 (26.7)

^a^ Victorian Certificate of Education. Equivalent to successful completion of high school.

Table 2 presents data relating to previous treatments participants had received for the osteoarthritis in their knee. Treatments included physiotherapy, massage and surgeries. Some participants had not received any other treatments prior to stem cell therapy (27% in the Expectations Group, 33% in the Experiences Group).

**TABLE 2:** Participants' previous treatments for osteoarthritis of the knee within the last 5 y (*N* = 30)

Treatment	Expectations group (*n* = 15)	Experiences group (*n* = 15)
No previous treatment *n* (%)	4 (26.7)	5 (33.3)
Self‐management^a^ *n* (%)	3 (20.0)	0 (0.0)
Physiotherapy *n* (%)	3 (20.0)	5 (33.3)
Injection therapy^b^ *n* (%)	2 (13.3)	5 (33.3)
Supplements^c^ *n* (%)	1 (6.7)	1 (6.7)
Massage therapy *n* (%)	2 (13.3)	1 (6.7)
Arthroscope *n* (%)	3 (20.0)	5 (33.3)
Other^d^ *n* (%)	3 (20.0)	4 (26.7)

^a^ Self‐management treatments included analgesic and anti‐inflammatory medications, measured exercises, strengthening and stretching.

^b^ Injection therapy treatment included platelet‐rich plasma (PRP), cortisone, hyaluronic acid (Synvisc) and Bowen therapy.

^c^ Supplements treatment included glucosamine chondroitin, fish oil, vitamin K2, turmeric, methylsulphonylmethane (MSM) and dimethyl sulphoxide (DMSO).

^d^ Other treatments included anterior cruciate ligament (ACL) reconstruction, magnesium rub, hydrotherapy, acupuncture, chiropractor, orthotics, osteopath, micro‐fracture and fluid aspiration.

### 3.1 Patient expectations group

Thematic analysis of the participant interviews in the Expectations Group (*n* = 15) revealed three main themes related to participants' expectations of stem cell therapy: (a) active involvement in the treatment, (b) treatment will improve symptoms and (c) benefits of treatment outweigh the risks.

#### 3.1.1 Active involvement in the treatment

Participants described the expectation of being actively involved in their treatment throughout their treatment trajectory. This commenced with the decision to undertake treatment. Most participants reported high levels of engagement in seeking treatment for their osteoarthritis.

I’d already done some research and I was looking at China and other places…because it [stem cell therapy] really isn’t accepted fully in Australia yet…as a standard procedure, where you can go get Medicare, so I tried to do any research development. (PARTICIPANT 10)

I went on the internet and start reading about it. I’ve done all the research and read all the comments people have put in about it. (PARTICIPANT 12)

The majority of participants displayed a comprehensive understanding of the treatment and expressed they felt well prepared for the treatment. This was achieved through their research in seeking stem cell therapy and the information provided by staff at the Stem Cell Clinic. Participants also felt comfortable to contact the Clinic if they had further specific questions or concerns:

I’d say that I feel fairly well informed, for the most part. If there was anything that I did want to be clarified, I would be in touch with them. (PARTICIPANT 4)

They gave me his number and said you know if you’ve got any concerns ring him night and day and you know, it doesn’t matter if it’s two in the morning you know he is happy to take your call if you have genuine concerns. (PARTICIPANT 9)

#### 3.1.2 Treatment will improve symptoms

Another expectation of participants was that the treatment will improve symptoms of their osteoarthritis. Specific symptoms described included increased mobility, reduction in pain and improved comfort. Some participants expressed this as a hope rather than an expectation of treatment:

I’m hopeful that my body will be in one of the 70% success rate and it actually grows cartilage. I’d like to work for a few more years and go through the day without any pain and walk with my wife. (PARTICIPANT 7)

I’m expecting, I’m hoping you know…hope rather than expect [that] it will maybe do enough to keep the knee going for another 15‐20 years. The residual sort of pain and discomfort in the leg would ease and disappear. (PARTICIPANT 15)

Hopefully, I’d like a successful result, where I’m pain free at night and able to walk distances without discomfort and to be able to bend my knees a lot easier. (PARTICIPANT 9)

#### 3.1.3 Benefits of treatment outweigh the risks

The final theme for participants' expectations of stem cell therapy was that the benefits of treatment outweigh the risks. Participants communicated the potential risks surrounding stem cell therapy in general terms and then more specifically expressed concerns of inefficacy, financial investment and possible infection.

You would be disappointed if it didn’t heal and I wasn’t pain free because of all the money I spent. (PARTICIPANT 2)

At the end of the day it’s only money and without that money…you might as well try and get the best out of it and help your life. (PARTICIPANT 5)

The only concern possible with stem cells is infection. (PARTICIPANT 10)

Participants considered stem cell therapy to be low risk compared with other traditional orthopaedic interventions because they were receiving their own stem cells and not an artificial device. Some participants that wanted to avoid a knee replacement viewed stem cell therapy to be a low risk alternative.

It should save a lot of people from having knee replacements. You don’t seem to get the full range of motion after a knee replacement. (PARTICIPANT 6)

Stem cell therapy is a less intrusive solution such as a knee replacement. (PARTICIPANT 11)

Participants expressed that while having the treatment may be a risk, they considered the risk worth the potential reward.

It could go really well, or it could not work at all. But that’s a risk I’m willing to take. (PARTICIPANT 4)

I would rather try this and risk being disappointed than do nothing and wait. (PARTICIPANT 8)

### 3.2 Patient experiences group

Thematic analysis of the participant interviews in the Experiences Group (*n* = 15) revealed three main themes related to participants' experiences of stem cell therapy: (a) symptoms of treatment, (b) satisfaction with treatment and (c) anticipation of further improvement.

#### 3.2.1 Symptoms of treatment

Throughout the treatment, participants encountered a range of different symptoms following the initial and/or secondary injections. Several participants voiced swelling as a symptom of their treatment but were aware this may be a result of the injections.

[clinic doctor A] had already advised me that [swelling after the 2^nd^ injection] was going to happen…he already told me and gave me, prescribed medication so that’s all under control. (PARTICIPANT 26)

I’d been sort of told up front that things were going to swell up and that sort of thing. (PARTICIPANT 28)

Some participants discussed how unexpected the location, severity and duration of the swelling was:

The ankle swelled up major, it was quite big and it hurt to kneel on it…it lasted for about a week or two, almost two weeks with that swelling. (PARTICIPANT 24)

The second injection, the second injection my whole leg blew up, my knee was stiff I couldn’t bend it, it was really awkward, trying to go to the toilet and everything was just so hard. (PARTICIPANT 21)

Separate unexpected difficulties arose for the small portion of participants that had micro‐fracture surgery prior to the initial injection which increased recovery time and interruption to daily life.

Because of the kids and the change in transportation my rehab over the last 12 months has been patchy so the first…4 or 5 months it was going well but then I lost…the opportunities to do a lot of rehab …I [still] have my duties as a parent to deal with and there was a lot of extra pressure on my wife. (PARTICIPANT 20)

Overall, participants felt they were adequately informed about the treatment including symptoms:

I feel I was well informed about the procedures, the risks, the flow of the events and also the outcomes so [clinic doctor A] briefed me through step by step throughout the journey. (PARTICIPANT 26)

Very [well informed]…there was a whole lot of stuff online you could read and [clinic doctor] gave me his, one of his scientific papers on the process. (PARTICIPANT 22)

#### 3.2.2 Satisfaction with treatment

Participants were asked how they felt their treatment went. Their responses described a general satisfaction with the treatment process.

I reckon it was good, I reckon it was successful. (PARTICIPANT 18)

It’s been successful. (PARTICIPANT 19)

Satisfaction was also expressed with participants stating they would repeat having the treatment as well as recommend it to others.

That’s all I do is recommend it……..Of course. Yes [I’d have stem cell treatment again]…because I, it’s been successful for me. (PARTICIPANT 24)

I certainly recommend someone trying it before having a knee replacement…I’d certainly suggest people have a look at it. (PARTICIPANT 28)

Some participants mentioned their satisfaction with respect to the positive treatment outcomes they had experienced. These included reduction in pain and increased functionality.

I’m doing things that used to potentially cause me pain but don’t cause me pain [now]…I wasn’t able to run without pain, I’m able to, like I run 3 kilometres…I wasn’t able to do that beforehand. (PARTICIPANT 27)

It’s going really well…I’m obviously walking around with minimal pain. (PARTICIPANT 25)

I can do things like you know, I can do things like drop slides, [high impact dance move] I can do you know like cha‐cha and sambas and rock and roll and quick step. (PARTICIPANT 24)

One participant expressed satisfaction with the treatment but disappointment in the outcome:

The job they did was excellent, the follow‐up and all. They sent me off with bandages and numbers to ring if anything happened. I just didn’t get the result I was hoping for. No real improvement. (PARTICIPANT 17)

#### 3.2.3 Anticipation of further improvement

The final theme for participants' experiences of stem cell therapy was the anticipation of further improvement. At the time of the interviews, all participants had completed treatment. However, participants referred to a belief that they would experience further improvements.

I would like to get back into high impact sport and I guess until I get word on when I can do that I’m going to be in a state of it’s not quite finished what it’s doing sort of…it does need more time. (PARTICIPANT 19)

The fact that they started getting better late in the piece makes me, I suppose, hopeful that they might continue to get better (PARTICIPANT 28)

I’m going back in 6 months’ time to get another MRI to see if there has been any further improvement. I’m hoping that it will continue to improve. And I know that they have had experience with other patients where it has taken a couple of years for it to, to really kick in and do its thing so I’m hoping I’m going to be one of the slow burners. (PARTICIPANT 23)

## 4 DISCUSSION AND CONCLUSION

### 4.1 Discussion

This is the first study to the authors' knowledge that explores stem cell therapy from the patient's perspective. Patients' expectations and experiences of stem cell therapy were explored. A person's overall experience of a treatment may be influenced by their expectations.^15^, ^19^ Patient expectations are defined as the belief that the occurrence of a particular event is likely.^37^ Expectations are influenced by prior experience, socio‐demographic factors, friends or family's scepticism/support and opinions, and therapeutic interactions including the process of informed consent.^38^, ^39^

#### 4.1.1 Participants expectations of stem cell therapy

Participants expected to have active involvement in their treatment. As stem cell therapy is a novel treatment for osteoarthritis, participants in this study were already actively seeking alternative treatments to traditional osteoarthritis treatments. This may explain participants' expectation of active involvement in their treatment. Research into non‐traditional therapies and self‐education is common for those who have already explored traditional avenues of treatment.^40^, ^41^ Active patient involvement in their care is beneficial for care delivery. Participants are more likely to be knowledgeable about treatment and play effective roles in decision making throughout the treatment process.^42^ Patients who are involved in their care and partner with health‐care professionals have shown a correlation with improved quality, safety, shorter hospital stays and increased patient satisfaction.^43^ It is vital that clinicians are prepared for and engage patients in their treatment in a way that is individualized to the person receiving the care.

Participants expected the treatment to improve their osteoarthritis symptoms. Specifically, they expected increased mobility, decreased pain and improved comfort. This research is situated in a context where the treatment is considered successful in up to 70% of people and requires a considerable financial investment.^44^ Patients receiving stem cell therapy for osteoarthritis either have a reduction in pain and/or functional limitations, and radiological improvement, or are non‐responsive with no change in any of their symptoms or condition.^29^, ^45^ For some participants, the word hope preceded an expectation of improved symptoms. Hope can be described as possessing a want or desire for a possible positive outcome, with the knowledge it may not eventuate.^46^ Expectations differ in that they are a predication or belief that something will happen.^47^ Hope expressed in this situation may relate to the participants awareness that the treatment is not effective in all patients and has been similarly voiced by patients in a study for the treatment of lower back pain.^39^

Financial cost, infection and inefficacy of treatment were factors participants reported to be risks associated with stem cell treatment. Despite these considerations, study participants believed that the benefits of stem cell treatment outweighed the potential risks. This is consistent with findings of a systematic review in which the majority of patients overestimated treatment benefits and underestimated harm.^38^ Even with high‐risk treatments, patients were willing to accept increased treatment risks to achieve improved function and disease control.^48^ The perceived reduced risks associated with stem cell therapy when compared to knee joint replacement may have contributed to participants motivations for deciding stem cell therapy.^16^, ^17^, ^18^ This highlights the need for clinicians to ensure accurate evidence‐based information is provided to patients in order to develop realistic expectations of treatment and its outcomes.

#### 4.1.2 Participants experiences of stem cell therapy

Patient experience is defined as the accumulation of all interactions that influence patient perception and view throughout the continuum of care.^49^ Collating this patient perspective enables early identification of problems in the care process with the potential to improve patient care.^50^, ^51^

The main symptom experienced by participants during treatment was swelling and pain. Despite expecting these, participants were surprised by the location, severity and duration of the swelling. This was highlighted in participants that received the micro‐fracture surgery prior to injection where the swelling and pain impacted their activities of daily living. Preparing patients to manage potential symptoms of treatment can ensure a smoother transition through recovery.^43^ Patient education is a key element of symptom management where patient understanding may empower the patient to actively manage their care.^43^, ^52^ Education enables patients to identify symptoms and adverse events in a timely manner because they are aware of what symptoms to expect^49^ and how to effectively manage the symptoms.^53^ The symptom experience of patients in this study suggests further targeted information and education is required to optimize symptom management.

Overall participants reported satisfaction with treatment and would recommend the treatment to others. Participants also articulated satisfaction with specific treatment outcomes including increased functionality and decreased pain. Unsurprisingly, increased functionality and reduced pain have shown to correlate with increased patient‐reported satisfaction following orthopaedic interventions.^37^, ^54^, ^55^ Interestingly, in a study measuring patient satisfaction following spinal surgery more than half the patients that experienced no improvement in treatment outcomes 12 months following treatment still reported satisfaction with the treatment.^56^ This was experienced by a participant in this study who expressed satisfaction with treatment despite no improvement. Satisfaction in this circumstance may have been influenced by factors outside of treatment outcomes.^57^, ^58^

Several participants described anticipation of further improvement in symptoms following the 12‐month period after the initial injection. This expectation is supported by evidence reporting incremental improvements in pain and functional scores years after the initial stem cell therapy treatment.^29^, ^59^, ^60^, ^61^, ^62^ Patients with positive expectations of treatment demonstrate better outcomes using both clinical measures^63^ and patient‐reported measures.^64^ In a study with participants undergoing knee arthroplasty, higher patient expectations predicted greater post‐operative improvement in patient‐reported outcomes. Met treatment expectations are associated with a more favourable patient experience.^39^, ^65^ Establishing patient expectations and their experiences is important for promoting patient‐centred care and has the potential to improve the quality and safety of care.

### 4.2 Strengths and limitations of the study

A major strength of this study was the exploratory, descriptive qualitative design. This design enabled insight into patients' expectations and experiences of stem cell therapy. While the study possesses strengths, it does have limitations. Due to the qualitative design and novel phenomenon, the data cannot be generalized to other populations or contexts. Additional research is required in this field to further explore this area and optimize the delivery of patient‐centred care.

### 4.3 Conclusion

The research project highlights the patient perspective through their expectations and experiences of stem cell therapy. It identifies the potential areas for improvement that could aid in patient's preparation and approach to the treatment, promoting patient‐centred care.

## CONFLICTS OF INTEREST

The authors report no conflicts of interest.

## Data Availability

Due to confidentiality and the nature of the consent obtained, the interview transcripts cannot be shared. For further information related to this data set, contact the corresponding author.
